# Association between delirium in the intensive care unit and subsequent neuropsychiatric disorders

**DOI:** 10.1186/s13054-020-03193-x

**Published:** 2020-07-31

**Authors:** Kyla N. Brown, Andrea Soo, Peter Faris, Scott B. Patten, Kirsten M. Fiest, Henry T. Stelfox

**Affiliations:** 1 Department of Community Health Sciences, University of Calgary, Calgary, Alberta Canada; 2 Alberta Health Services, Calgary, Alberta Canada; 3 Department of Critical Care Medicine, University of Calgary, 3134 Hospital Drive, Calgary, Alberta T2N 2T9 Canada; 4 O’Brien Institute for Public Health, University of Calgary, Calgary, Alberta Canada; 5 Hotchkiss Brain Institute, University of Calgary, Calgary, Alberta Canada; 6 Department of Psychiatry, University of Calgary, Calgary, Alberta Canada

**Keywords:** Cohort studies, Mental health, Cognition disorders, Critical care, Adult

## Abstract

**Background:**

Patients in the intensive care unit (ICU) are known to be at increased risk of developing delirium, but the risk of subsequent neuropsychiatric disorders is unclear. We therefore sought to examine the association between the presence of delirium in the ICU and incident neuropsychiatric disorders (including depressive, anxiety, trauma-and-stressor-related, and neurocognitive disorders) post-ICU stay among adult medical-surgical ICU patients.

**Methods:**

Retrospective cohort study utilizing clinical and administrative data from both inpatient and outpatient healthcare visits to identify the ICU cohort and diagnostic information 5 years prior to and 1 year post-ICU stay. Patients ≥ 18 years of age admitted to one of 14 medical-surgical ICUs across Alberta, Canada, January 1, 2014–June 30, 2016, and survived to hospital discharge were included. The main outcome of interest was a new diagnosis of any neuropsychiatric disorder 1 year post-ICU stay. The exposure variable was delirium during the ICU stay identified through any positive delirium screen by the Intensive Care Unit Delirium Screening Checklist (ICDSC) during the ICU stay.

**Results:**

Of 16,005 unique patients with at least one ICU admission, 4033 patients were included in the study of which 1792 (44%) experienced delirium during their ICU stay. The overall cumulative incidence of any neuropsychiatric disorder during the subsequent year was 19.7% for ICU patients. After adjusting for hospital characteristics using log-binomial regression, patients with delirium during the ICU stay had a risk ratio (RR) of 1.14 (95% confidence interval [CI] 0.98–1.33) of developing any neuropsychiatric disorder within 1 year post-ICU compared to those who did not experience delirium. Delirium was significantly associated with neurocognitive disorders (RR 1.59, 95% CI 1.08–2.35), but not depressive disorders (RR 1.16, 95% CI 0.92–1.45), anxiety (RR 1.16, 95% CI 0.92–1.47), and trauma-and-stressor-related (RR 0.82, 95% CI 0.53–1.28) disorders.

**Conclusions:**

The diagnosis of new onset of neurocognitive disorders is associated with ICU-acquired delirium. In this study, significant associations were not observed for depressive, anxiety, and trauma-and-stressor-related disorders.

## Background

Delirium is an acute state characterized by an onset of confusion and decline in cognitive ability often occurring in hospitalized patients [1–5]. The prevalence of delirium in the intensive care unit (ICU) ranges from 20 to 80% [1]. Delirium is associated with an increased length of ICU and hospital stay [6] and mortality [7, 8]. A number of studies have suggested that delirium-induced stress response may predispose patients to the development of neuropsychiatric disorders [3–5, 9, 10], in particular dementia [11]. The hypothesized mechanisms for this relationship include biological and chemical changes in the brain as a response to stress and inflammation [12], disturbances in circadian rhythm [12, 13], and the use of sedatives, memory deficits, and treatment-related trauma [14, 15].

Risk factors for delirium have similarly been implicated in the psychiatric morbidity of critically ill patients post-stay [14, 15]. A number of studies suggest that patients admitted to the ICU are at an increased risk of subsequently developing neuropsychiatric disorders [16–18]. Neuropsychiatric disorders can include depressive, anxiety, trauma-and-stressor-related, and neurocognitive disorders [16–18]. However, there are few and limited population-based research studies using physician-diagnosed codes to examine the association between delirium in the ICU and subsequent neuropsychiatric disorders post-stay. Given the high prevalence of delirium, it is important to understand whether these ICU patients are at increased risk of neuropsychiatric disorders relative to patients who did not experience delirium [19–21].

The aim of the current study was to evaluate the association between delirium in the ICU and a new diagnosis of any neuropsychiatric disorder in adult medical-surgical ICU patients during 1 year post-ICU stay. We hypothesized that patients with delirium in the ICU would be at an increased risk of developing neuropsychiatric disorders during the 1-year follow-up period.

## Methods

This retrospective cohort study was conducted and reported in accordance with recommendations of the Strengthening the Reporting of Observational Studies in Epidemiology (STROBE) statement [22]. The Conjoint Health Research Ethics Board at the University of Calgary approved this study (REB17-0389).

### Study cohort

We identified all adult ICU patients (≥ 18 years) who were admitted to one of the 14 medical-surgical ICU across Alberta, Canada (population 4.2 million people), between January 1, 2014, and June 30, 2016 [23]. Data was collected 5 years prior to an individual ICU admission and up to 1 year after hospital discharge to identify individuals who had a healthcare visit for a diagnosis of a neuropsychiatric disorder. Patients were excluded if their ICU stay was less than 24 h, if they died in ICU, if they did not have any delirium assessment in ICU, if they did not survive to hospital discharge, if their ICU admission did not link with hospital admission data within a 2-h window, or if their home was located outside of Alberta (to allow for complete follow-up). In the case of multiple ICU admissions during the study period, the patient’s first eligible ICU admission was used as the primary admission. Patients with an inpatient or outpatient healthcare visit for which any neuropsychiatric disorder (i.e., depressive, anxiety, trauma-and-stressor, and neurocognitive disorders) was listed as a post-admit comorbidity or a secondary diagnosis in the 5 years prior to and during their primary ICU admission were excluded (i.e., diagnostic codes suggestive of a pre-existing neuropsychiatric disorder). Hospital discharge dates ranged from January 9, 2014, to March 13, 2017. Patients were followed up until 1 year post-hospital discharge, death, or study end (October 31, 2017), whichever occurred first.

### Data sources

We used clinical and administrative databases from Alberta Health Services and Alberta Health (Discharge Abstracts Database [DAD], National Ambulatory Care Reporting System [NACRS], Physician Claims, Vital Statistics, and eCritical Tracer Database), previously successfully used in research studies [24, 25]. eCritical Tracer is a bedside electronic medical record that prospectively captures demographic, clinical, and patient outcomes for all patients admitted to an Alberta ICU [26]. The DAD captures patients who received hospital-based care and has up to 25 *Canadian Enhancement of International Statistical Classification of Diseases*, *10th Revision*, fields for diagnostic codes. The NACRS captures data for all hospital-based and community-based emergency or ambulatory care and has up to 10 *Canadian Enhancement of International Statistical Classification of Diseases*, *10th Revision*, fields for diagnostic codes. Physician Claims captures patients who received care from an outpatient clinic (primary or specialty) and has up to 3 *Canadian Enhancement of International Statistical Classification of Diseases*, *9th Revision*, fields for diagnostic codes. Vital Statistics data is provided to Alberta Health Services from Alberta Health. All deaths occurring in Alberta must be registered with Alberta Vital Statistics. The DAD, NACRS, and Physician Claims were available until October 31, 2017, and Vital Statistics were available until December 31, 2017, at the time of data extraction.

### Delirium

The primary exposure was ever having delirium at any point during the ICU stay. Delirium was measured using the validated Intensive Care Delirium Screening Checklist (ICDSC) [27], which is documented as part of standard of care by the bedside nurse once every nursing shift (i.e., twice a day) [28]. The checklist is scored out of eight categories that include the level of consciousness, inattention, disorientation, hallucinations/delusions/psychosis, psychomotor agitation, inappropriate speech or mood, sleep wake/cycle disturbance, and symptom fluctuation [29]. Each category is coded as present (i.e., a score of 1) if the patient meets the criteria listed, for a maximum score of 8. Patients who scored ≥ 4 on the ICDSC at any time during the ICU stay were categorized as ever having delirium and those with all of their ICDSC scores < 4 on all ratings were categorized as never having delirium. The ICDSC has a sensitivity of 99% and a specificity of 64% in a medical-surgical ICU patient population [29]. Sensitivity analyses were conducted to determine whether there was a dose-response according to the duration of delirium in the ICU (one calendar day of delirium vs. two or more calendar days of delirium). One calendar day of delirium was determined by having at least one ICDSC score of ≥ 4 on that calendar day.

### Neuropsychiatric disorders

Neuropsychiatric disorders identified in previous systematic reviews to potentially be associated with delirium were included as outcome measures [30–33], including depressive (such as major depressive disorders), anxiety (such as generalized anxiety disorders), trauma-and-stressor-related (such as acute stress disorders or post-traumatic stress disorder), and neurocognitive (such as dementia, respective symptoms for neurocognitive disorders include difficulty performing tasks in high stimuli environment, forgetting words, and experiencing personality changes [34]) disorders. Neuropsychiatric disorders were identified through physician-diagnosed ICD 9/10 codes that were captured in the DAD, NACRS, and Physician Claims databases (see Additional file 1). Validated coding algorithms were used to identify depressive [35] and anxiety [36] disorders. A neuropsychiatrist and psychiatrist developed coding algorithms for trauma-and-stressor-related and neurocognitive disorders as there are currently no published validated coding algorithms for these disorders. The two clinicians independently reviewed all DSM-5 codes as well as previous studies reporting these disorders identifying those ICD 9/10 codes for trauma-and-stressor-related and neurocognitive disorders. Disagreements were resolved through discussion. The primary outcome was a healthcare visit (e.g., hospital stay, emergency or ambulatory, and outpatient clinic) that was a post-admit comorbidity or a secondary diagnosis of a neuropsychiatric disorder (i.e., depressive, anxiety, trauma-and-stressor-related, and neurocognitive disorders). Neuropsychiatric disorders were examined together to identify individuals who were diagnosed with *any* of the four neuropsychiatric disorders and then for each disorder separately.

### Covariates

Characteristics potentially associated with delirium during the ICU stay and neuropsychiatric disorders at a 1-year follow-up were identified a priori [37, 38]*.* Characteristics included age, sex, ICU admission reason (medical, surgical, neurological, trauma), Acute Physiology and Chronic Health Evaluation (APACHE) II score, Charlson Comorbidity Index (CCI), Glasgow Coma Scale (GCS), transfer delay ≥ 24 h, invasive mechanical ventilation (yes/no), non-invasive mechanical ventilation (yes/no), continuous renal replacement therapy (yes/no), vasoactive medications (yes/no), ICU size (categorized as < 20 beds vs. ≥ 20 beds), teaching hospital (yes/no), ICU length of stay (categorized as < 7 days vs. ≥ 7 days), and last Sequential Organ Failure Assessment (SOFA) score.

### Statistical analysis

Descriptive statistics for demographic characteristics and covariates were examined using median (interquartile range [IQR]) for continuous variables and frequencies (with percentages) for categorical variables. Group comparisons were made using the Mann-Whitney *U* test for continuous non-normally distributed variables and chi-squared tests for binary variables. The cumulative incidence of any and each neuropsychiatric disorder was calculated by identifying the number of new cases of the neuropsychiatric disorder within 1 year (the numerator) divided by the total number of patients at risk of developing the disorder (the denominator). The incidence density of any and each neuropsychiatric disorder was calculated by identifying the number of new cases of the neuropsychiatric disorder within 1 year (the numerator) divided by the total number of person years for patients at risk of developing the disorder (the denominator). For patients who did not survive to 1 year post-ICU stay, time from hospital discharge to death was taken as time at risk.

Associations between delirium in the ICU and new neuropsychiatric disorders were assessed using log-binominal regression analyses (in which exponentiated regression coefficients represent risk ratios). Supplementary analyses were also conducted for each disorder separately (depressive, anxiety, trauma-and-stressor-related, and neurocognitive disorders) using log-binomial regression analyses. We adjusted all models for all covariates previously identified. Analyses were conducted in STATA version 14 (StataCorp LP; College Station, TX, USA) and R, version 3.5.1 (The R Project for Statistical Computing, http://www.r-project.org/).

## Results

### Study population

**Fig. 1:**
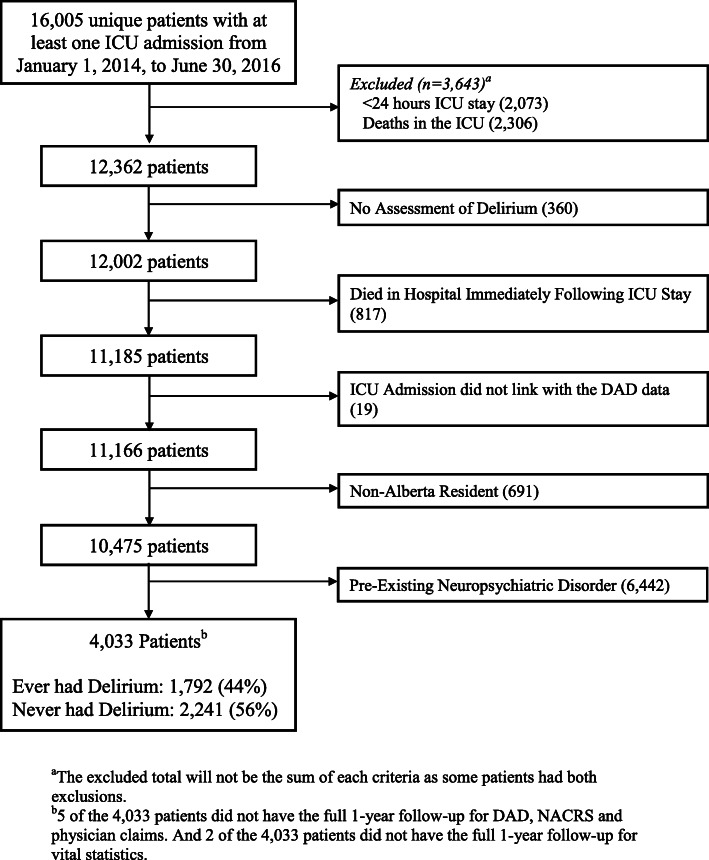
Study flow chart

From January 1, 2014, to June 30, 2016, there were 16,005 unique patients from the 14 study ICUs with at least one ICU stay. Of these ICU admissions, there were 11,166 unique ICU patients potentially eligible for inclusion in the study (exclusion reasons included ICU stay < 24 h, death in ICU, no delirium assessment, died in hospital following first ICU admission, patient’s ICU admission did not link with the DAD admission data within a 2-h window, non-Albertan patients). Of those, 6442 patients had a healthcare visit within the previous 5 years that included a diagnosis of a neuropsychiatric disorder, leaving 4033 patients who satisfied the inclusion criteria (Fig. 1). The baseline characteristics of patients with and without pre-existing neuropsychiatric disorders are outlined in Additional file 2. Among the 4033 patients included in the study, 475 died (12%) within the 1-year follow-up.

Patient and hospital characteristics of the primary study cohort are summarized in Table 1. The overall prevalence of delirium during the ICU stay was 44% (95% confidence interval [CI] 43–46%) (*n* = 1792). The median age of ICU patients in this study was 60 years (IQR 46–71 years), 68% (*n* = 2744) were male, the most common reason for an ICU admission was medical (e.g., pneumonia, cardiac arrest, sepsis, etc.), and the median length of ICU stay was 4.1 days (IQR 2.3–7.6 days). The median Charlson Comorbidity Index was 1 (IQR 0–3), median SOFA score upon ICU admission was 6 (IQR 3–9), and median APACHE II score upon ICU admission was 18 (IQR 13–24).

**Table 1:** Patient and hospital characteristics during the intensive care unit for those who survived to hospital discharge

	Total (***n*** = 4033)	Delirium^**a**^		***p*** value
Characteristic		Delirium (***n*** = 1799)	No delirium (***n*** = 2234)	
**Patient characteristics on ICU admission**				
Age, median (IQR) years	60 (46–71)	60 (46–70)	60 (46–71)	0.85
Male sex, no. (%)	2744 (68.0)	1278 (71.0)	1466 (65.6)	< 0.001
Reason for ICU admission, no. (%)^b^				
Medical	2172 (54.9)	939 (52.8)	1233 (56.7)	< 0.001
Surgical	1221 (30.9)	449 (25.2)	772 (35.5)	
Neurological	205 (5.2)	143 (8.0)	62 (2.9)	
Trauma	356 (9.0)	249 (14.0)	107 (4.9)	
Comorbidities, no. (%)^c^				
AIDS	6 (0.2)	1 (0.1)	5 (0.2)	0.24
Acute myocardial infarction	270 (6.7)	140 (7.8)	130 (5.8)	0.011
Cancer	587 (14.6)	210 (11.7)	377 (16.8)	< 0.001
Cerebrovascular disease	201 (5.0)	143 (8.0)	58 (2.6)	< 0.001
Congestive heart failure	435 (10.8)	206 (11.5)	229 (10.2)	0.19
Chronic obstructive pulmonary disease	699 (17.3)	321 (17.9)	378 (16.9)	0.38
Diabetes	518 (12.8)	230 (12.8)	288 (12.9)	0.99
Diabetes + complications	694 (17.2)	324 (18.1)	370 (16.5)	0.19
Hemiplegia or paraplegia	90 (2.2)	53 (3.0)	37 (1.7)	0.005
Metastatic cancer	167 (4.1)	57 (3.2)	110 (4.9)	0.006
Mild liver disease	196 (4.9)	108 (6.0)	88 (3.9)	0.002
Moderate/severe liver disease	104 (2.6)	62 (3.5)	42 (1.9)	0.002
Peptic ulcer disease	153 (3.8)	72 (4.0)	81 (3.6)	0.51
Peripheral vascular disease	228 (5.7)	106 (5.9)	122 (5.4)	0.52
Renal disease	207 (5.1)	97 (5.4)	110 (4.9)	0.47
Rheumatoid disease	61 (1.5)	29 (1.6)	32 (1.4)	0.62
Charlson Comorbidity Score, median (IQR)	1 (0–3)	1 (0–2)	1 (0–3)	0.17
SOFA score on ICU admission, median (IQR)	6 (3–9)	7 (5–10)	5 (3–7)	< 0.001
APACHE II score on ICU admission, median (IQR)	18 (13–23)	20 (15–26)	16 (11–21)	< 0.001
Glasgow Coma Scale score on ICU admission, median (IQR)	15 (13–15)	14 (11–15)	15 (15–15)	< 0.001
**Interventions received in ICU**				
Invasive mechanical ventilation	2535 (62.9)	1463 (81.3)	1072 (48.0)	< 0.001
Non-invasive mechanical ventilation	569 (14.1)	276 (15.3)	293 (13.1)	0.043
Continuous renal replacement therapy	181 (4.5)	145 (8.1)	36 (1.6)	< 0.001
Vasoactive medications	1770 (43.9)	1035 (57.5)	735 (32.9)	< 0.001
**Hospital characteristics**				
≥ 20 ICU beds, no. (%)	2161 (53.6)	1084 (60.3)	1077 (48.2)	< 0.001
≥ 600 hospital beds, no. (%)	2404 (59.6)	1186 (65.9)	1218 (54.5)	< 0.001
Teaching hospital, no. (%)	3304 (81.9)	1571 (87.3)	1733 (77.6)	< 0.001
≥ 24 h in ICU after ready for ICU discharge	1505 (37.3)	695 (38.6)	810 (36.3)	0.12
**Patient characteristics on ICU discharge**				
Length of ICU stay, median (IQR) days	4.1 (2.3–7.6)	6.6 (3.8–12.4)	2.9 (1.9–4.8)	< 0.001

*Abbreviations*: *AIDS* Auto-immune deficiency syndrome, *APACHE II score* Acute Physiology and Chronic Health Evaluation II Score, *ICU* Intensive care unit, *SOFA* Sequential Organ Failure Assessment

^a^Patients who scored positive for delirium by the Intensive Care Unit Delirium Screening Checklist (ICDSC) of a score greater than or equal to 4 at any time during the ICU were categorized as ever having delirium

^b^Mutually exclusive categories; 77 patients missing data

^c^Non-mutually exclusive categories

### Incidence of neuropsychiatric disorders

During the 1-year follow-up period, 794 patients (19.7%, 95% CI 18.5–20.9%) had a healthcare visit for a newly diagnosed neuropsychiatric disorder. The cumulative incidences of depressive, anxiety, trauma-and-stressor-related, and neurocognitive disorders were 10.1% (95% CI 9.2–11.1%), 8.8% (95% CI 8.0–9.7%), 2.9% (95% CI 2.3–3.4%), and 3.5% (95% CI 2.9–4.1%), respectively. The incidence density for those who developed any neuropsychiatric disorder during the 1-year follow-up was 213 (95% CI 200–226) per 1000 person years. The incidence densities of depressive, anxiety, trauma-and-stressor-related, and neurocognitive disorders were 110 (95% CI 100–120) per 1000 person years, 96 (95% CI 86–105) per 1000 person years, 31 (95% CI 25–36) per 1000 person years, and 38 (95% CI 32–44) per 1000 person years, respectively.

### Delirium and neuropsychiatric disorders

Patients who ever had delirium in the ICU had 1.14 (95% CI 0.98–1.33) times the risk of developing a neuropsychiatric disorder within 1 year compared to those who never had delirium in the ICU (Table 2). The adjusted risk ratios of depressive, anxiety, trauma-and-stressor-related, and neurocognitive disorders were 1.16 (95% CI 0.92–1.45), 1.16 (95% CI 0.92–1.47), 0.82 (95% CI 0.53–1.28), and 1.59 (95% CI 1.08–2.35), respectively, for those with delirium compared to those without delirium in the ICU (Table 2, see Additional file 3 for unadjusted estimates and Additional file 4 for adjusted estimates). A sensitivity analysis using duration of delirium, defined as (1) never having delirium, (2) one calendar day of delirium, and (3) two or more calendar days of delirium, showed similar adjusted risk ratios for any neuropsychiatric disorder, as well as each specific neuropsychiatric disorder of interest except neurocognitive disorders (see Additional file 5). Patients with two or more calendar days of delirium in the ICU had 2.14 (95% CI 1.40–3.28) times the risk of developing a neurocognitive disorder within the 1 year compared to those who never had delirium, whereas patients with one calendar day of delirium did not have a significantly different risk of developing a neurocognitive disorder within the 1 year compared to those who never had delirium (see Additional file 5).

**Table 2:** Association between delirium during the intensive care unit and subsequent neuropsychiatric disorders

	Risk ratio (95% confidence interval)^**a**^	
Outcome	Crude	Adjusted^**b**^
Any neuropsychiatric disorder	1.36 (1.20–1.54)	1.14 (0.98–1.33)
Depressive	1.45 (1.20–1.74)	1.16 (0.92–1.45)
Anxiety	1.31 (1.08–1.60)	1.16 (0.92–1.47)
Trauma-and-stressor related	1.14 (0.79–1.63)	0.82 (0.53–1.28)
Neurocognitive	2.19 (1.57–3.09)	1.59 (1.08–2.35)

^a^Reference group is “never” had ICU delirium

^b^Adjusted for age, sex, ICU admission reason (medical, surgical, neurological, trauma), APACHE II score, Charlson Comorbidity score, Glasgow Coma Scale, transfer delay, ≥ 24 h, invasive mechanical ventilation (yes/no), continuous renal replacement therapy (yes/no), and vasoactive medications (yes/no), ≥ 20 ICU beds, teaching hospital (yes/no), ICU length of stay (categorized as < 7 days vs. ≥ 7 days), and last SOFA score

## Discussion

This retrospective cohort study examined the association between delirium in the ICU and subsequent healthcare visits for a neuropsychiatric disorder, captured in both inpatient and outpatient settings. There was a high prevalence of delirium in the study cohort, which is consistent with the existing literature on the prevalence of delirium in the ICU [1, 39]. In addition, a high cumulative incidence of neuropsychiatric disorders was observed during the 1-year follow-up. Patients who ever had delirium during the ICU were at a significantly increased risk of developing neurocognitive disorders at 1-year follow-up, specifically those with delirium for two or more days.

There is a growing awareness of neuropsychiatric disorders following an ICU stay [20, 40]. A study by Paparrigopoulos et al. [41] of 48 ICU survivors found that patients with pre-existing neuropsychiatric disorders are at an increased risk of developing subsequent disorders post-ICU stay. Patients without pre-existing disorders also face a high risk of developing new neuropsychiatric disorders post-ICU stay as shown from this study. This is in addition to the general population prevalence of depression reported to be 4.7% in 2012 [42], and the general population estimate of a lifetime anxiety disorder diagnosis ranges from 14.6 [43] to 24.9% [44]. A study by Granja et al. [15] of 464 ICU patients and Chahraoui et al. [45] of 20 ICU patients examined risk factors for neuropsychiatric disorders post-ICU stay, suggesting that recollection (e.g., those who do not remember their stay) and negative memories (e.g., persistent nightmares) associated with an ICU stay often predicted a worse quality of life and risk for neuropsychiatric sequelae. Additional risk factors for ICU patients developing neuropsychiatric disorders, specifically depression, stem from factors such as age, gender, the nature of therapies provided in the ICU, length of stay, and diagnosis of sepsis [46, 47]. The implications of a growing awareness of neuropsychiatric disorders following an ICU stay are that care plans for patients post-ICU may benefit from surveillance and targeted therapies.

Our study showed a significant association between delirium in the ICU and a subsequent diagnosis of a neurocognitive disorder. However, our study did not find significant associations for depressive, anxiety, and trauma-and-stressor-related disorders, potentially due to the comorbid relationship between depression and anxiety, as depressive disorders may present more prominent symptoms leading to the misclassification of anxiety disorders as depressive disorders [48]. Furthermore, individuals may present with symptoms of depression and not be formally diagnosed [34]. Given the high burden and risk of neuropsychiatric disorders following an ICU stay, further evaluations of psychiatric health and cognitive-based interventions should be a priority for ICU patients in follow-up clinics [49]. Critical care physicians should be aware of the potential long-term ramifications of an ICU stay, in addition to the physical health of ICU patients [50]. Implementation of delirium screening tools during the ICU stay may lead to earlier detection and treatment and potentially help to mitigate the negative effects of delirium and the associated risk of subsequent neurocognitive disorders [13].

Strengths of this study include the large sample size and population-based nature of the data, as well as the ability to control for a number of risk factors for delirium and neuropsychiatric disorders. Data were also available for pre-existing neuropsychiatric disorders. Moreover, this study was able to utilize a provincial, validated delirium identification tool [28]. Our study findings have some limitations. First, trauma-and-stressor-related disorders and neurocognitive disorders did not have a validated coding scheme; therefore, the code list developed may not include all the ICD 9/10 codes related to each disorder, resulting in possible underestimates of the incidence and risk ratio. Second, our study sample may have underestimated the incidence of neuropsychiatric disorders as we only captured healthcare visits to medical professionals, potentially missing visits to other therapists. This measurement approach is likely to identify patients with more severe neuropsychiatric disorders and provide more specific (and less sensitive) estimates. A subset of patients may also choose not to seek help due to mental health-associated stigma [51] or have difficulty identifying symptoms [52]. Third, the follow-up period post-ICU stay was only 1 year in length, which may not be long enough for some neuropsychiatric disorders (e.g., dementia) to manifest and be diagnosed. Fourth, our study excluded patients with previously diagnosed neuropsychiatric disorders and is therefore likely to provide a conservative estimate of the association between delirium and new or worsening neuropsychiatric disorders. Fifth, we did not capture sedative medication or alcohol exposure, which may lead to residual confounding.

## Conclusions

Our data suggest that patients who experience delirium during the ICU are at increased risk of developing a neurocognitive disorder in the 1 year following their ICU stay. This risk appears to be dose dependent. Significant associations with depressive, anxiety, and trauma-and-stressor-related disorders were not observed, but the confidence intervals associated with risk ratios for these disorders do not necessarily preclude the existence of clinically significant associations between delirium and these conditions. Future clinical and research efforts should focus on early detection and management of delirium to mitigate the potential development of neurocognitive disorders post-ICU stay. Consideration should be given to monitoring ICU patients with delirium for long-term neurocognitive disorders.

## Supplementary information

**Additional file 1.** Frequency of Diagnostic Codes for Each Neuropsychiatric Disorder Per Visit Per Database for All Patient Data.: The data presented in additional file 1 present frequency of diagnostic codes for each neuropsychiatric disorder per visit per database (DAD, NACRS, and Physician Claims) for all patient data.

**Additional file 2.** Characteristics of Those With and Without Pre-Existing Neuropsychiatric Disorders. The data presented in additional file 2 compare characteristics of patients with and without pre-existing neuropsychiatric disorders from five years prior to ICU admission.

**Additional file 3.** Unadjusted Risk Ratios for Delirium, Patient Characteristics and Neuropsychiatric Disorders. The data presented in additional file 3 present detailed unadjusted risk ratios for delirium, patient characteristics and each neuropsychiatric disorder.

**Additional file 4.** Adjusted Models for Delirium and Neuropsychiatric Disorders. The data presented in additional file 4 present detailed models for delirium and each neuropsychiatric disorder.

**Additional file 5.** Sensitivity Analyses Between Delirium and No Delirium/one calendar day/two or more calendar days of Delirium in the Intensive Care Unit. The data presented in additional file 5 is the outputs of a sensitivity analysis between delirium and no delirium/one calendar day/two or more calendar days of delirium in the ICU.

## Data Availability

The datasets generated and analyzed during the current study are not publicly available due to identifiable information of patients but are available from the corresponding author on reasonable request.

## Abbreviations

APACHE II: Acute Physiology and Chronic Health Evaluation II
CCI: Charlson Comorbidity Index
GCS: Glasgow Coma Scale
SOFA: Sequential Organ Failure Assessment
CI: Confidence Interval
IQR: Inter-Quartile Range
ICDSC: Intensive Care Delirium Screening Checklist
AIDS: Aquired Immuno-Deficiency Syndrome
DAD: Discharge Abstracts Database
ICU: Intensive care unit
NACRS: National Ambulatory Care Reporting System
STROBE: Strengthening the Reporting of Observational Studies in Epidemiology
