# Supplementary material for: Association between delirium in the intensive care unit and subsequent neuropsychiatric disorders
Source: Crit Care. 2020 Jul 31;24:476. doi: 10.1186/s13054-020-03193-x (PMC7393876; doi:10.1186/s13054-020-03193-x)
Supplement: Supplementary file 1 — Additional file 1. Frequency of Diagnostic Codes for Each Neuropsychiatric Disorder Per Visit Per Database for All Patient Data.: The data presented in additional file 1 present frequency of diagnostic codes for each neuropsychiatric disorder per visit per database (DAD, NACRS, and Physician Claims) for all patient data. [file 13054_2020_3193_MOESM1_ESM.docx]

Additional File 1. Frequency of Diagnostic Codes for Each Neuropsychiatric Disorder Per Visit Per Database for All Patient Data

| **DISCHARGE ABSTRACTS DATABASE** | | | | | |
| --- | --- | --- | --- | --- | --- |
| **ICD-10** | **Definition** | **Total**  **No. (%)** | **Before ICU**  **No. (%)** | **During ICU**  **No. (%)** | **After ICU**  **No. (%)** |
| **Depressive** | | **4,327** | **2,446** | **868** | **1,013** |
| F31.3 | Bipolar affective disorder, current episode mild or moderate depression | 25 (0.6%) | 16 (0.7%) | 3 (0.4%) | 6 (0.6%) |
| F31.4 | Bipolar affective disorder, current episode severe depression without psychotic symptoms | 33 (0.8%) | 13 (0.5%) | 8 (0.9%) | 12 (1.2%) |
| F31.5 | Bipolar affective disorder, current episode severe depression with psychotic symptoms | 12 (0.3%) | 5 (0.2%) | 2 (0.2%) | 5 (0.5%) |
| F31.6 | Bipolar affective disorder, current episode mixed | 28 (0.7%) | 25 (1.0%) | - | 3 (0.3%) |
| F31.9 | Bipolar affective disorder, current episode unspecified | 631 (14.6%) | 347 (14.2%) | 131 (15.1%) | 153 (15.1%) |
| F32.0 | Mild depressive episode | 2 (0.1%) | 1 (0.0%) | - | 1 (0.1%) |
| F32.1 | Moderate depressive episode | 1 (0.0%) | 1 (0.0%) | - | - |
| F32.2 | Severe depressive episode without psychotic symptoms | 426 (9.9%) | 200 (8.2%) | 105 (12.1%) | 121 (11.9%) |
| F32.3 | Severe depressive episode with psychotic symptoms | 49 (1.1%) | 34 (1.4%) | 8 (0.9%) | 7 (0.7%) |
| F32.4 | Depressive disorder, single episode, in partial remission | - | - | - | - |
| F32.5 | Depressive disorder, single episode, in the full remission | - | - | - | - |
| F32.8 | Other depressive episodes | 25 (0.6%) | 13 (0.5%) | 4 (0.5%) | 8 (0.8%) |
| F32.9 | Depressive episode unspecified | 2,121 (49.0%) | 1,210 (49.5%) | 395 (45.5%) | 516 (50.9%) |
| F33.0 | Recurrent depressive disorder, current episode mild | 4 (0.1%) | 2 (0.1%) | - | 2 (0.2%) |
| F33.1 | Recurrent depressive disorder, current episode moderate | 22 (0.5%) | 18 (0.7%) | 3 (0.4%) | 1 (0.1%) |
| F33.2 | Recurrent depressive disorder, current episode severe without psychotic symptoms | 72 (1.7%) | 47 (1.9%) | 14 (1.6%) | 11 (1.1%) |
| F33.3 | Recurrent depressive disorder, current episode severe with psychotic symptoms | 10 (0.2%) | 5 (0.2%) | - | 5 (0.5%) |
| F33.4 | Recurrent depressive disorder, currently in remission | 10 (0.2%) | 6 (0.3%) | - | 4 (0.4%) |
| F33.8 | Recurrent depressive disorder, other | 9 (0.2%) | 2 (0.1%) | 4 (0.5%) | 3 (0.3%) |
| F33.9 | Recurrent depressive disorder, unspecified | 115 (2.7%) | 72 (2.9%) | 27 (3.1%) | 16 (1.6%) |
| F34.1 | Dysthymia | 141 (3.3%) | 103 (4.2%) | 23 (2.7%) | 15 (1.5%) |
| F34.8 | Other persistent mood disorder | 1 (0.0%) | 1 (0.0%) | - | - |
| F34.9 | Persistent mood disorders, unspecified | - | - | - | - |
| F38.0 | Other single mood disorders | - | - | - | - |
| F38.1 | Other recurrent mood disorders | 8 (0.2%) | 5 (0.2%) | 2 (0.2%) | 1 (0.1%) |
| F38.8 | Other specified mood disorders | 20 (0.5%) | 15 (0.6%) | 4 (0.5%) | 1 (0.1%) |
| F39 | Unspecified mood disorder | 132 (3.1%) | 79 (3.2%) | 28 (3.2%) | 25 (2.5%) |
| F41.2 | Mixed anxiety and depressive disorder | 624 (14.4%) | 331 (13.5%) | 155 (17.9%) | 138 (13.6%) |
| F99 | Mental disorder, not elsewhere specified | 7 (0.2%) | 3 (0.1%) | 1 (0.1%) | 3 (0.3%) |
| **Anxiety** | | **1,344** | **693** | **332** | **319** |
| F40.0 | Agoraphobia | 17 (1.3%) | 9 (1.3%) | 5 (1.5%) | 3 (0.9%) |
| F40.1 | Social Phobias | 13 (1.0%) | 8 (1.2%) | 3 (0.9%) | 2 (0.6%) |
| F40.2 | Specific (isolated) Phobia | 5 (0.4%) | 2 (0.3%) | - | 3 (0.9%) |
| F40.8 | Other Phobic Anxiety Disorder | 2 (0.2%) | 2 (0.3%) | - | - |
| F40.9 | Phobic Anxiety Disorder, Unspecified | - | - | - | - |
| F41.0 | Panic Disorder | 89 (6.6%) | 50 (7.2%) | 16 (4.8%) | 23 (7.2%) |
| F41.1 | Generalized Anxiety Disorder | 248 (18.5%) | 122 (17.6%) | 74 (22.3%) | 52 (16.3%) |
| F41.8 | Other Specific Anxiety Disorder | 36 (2.7%) | 19 (2.7%) | 11 (3.3%) | 6 (1.9%) |
| F41.9 | Unspecified Anxiety Disorder | 984 (73.2%) | 507 (73.2%) | 231 (69.6%) | 246 (77.1%) |
| **Trauma-and-Stressor Related** | | **998** | **480** | **246** | **272** |
| F43.0 | Acute stress disorder | 43 (4.3%) | 21 (4.4%) | 15 (6.1%) | 7 (2.6%) |
| F43.1 | Post-traumatic stress disorder | 242 (24.3%) | 117 (24.4%) | 57 (23.2%) | 68 (25.0%) |
| F43.2 | Adjustment disorders | 731 (73.3%) | 347 (72.3%) | 181 (73.6%) | 203 (74.6%) |
| F43.8 | Other specified trauma-and stressor related disorder | 4 (0.4%) | - | 2 (0.8%) | 2 (0.7%) |
| F43.9 | Unspecified trauma-and stressor related disorder | 14 (1.4%) | 10 (2.1%) | 3 (1.2%) | 1 (0.4%) |
| **Neurocognitive** | | **2,635** | **1,097** | **889** | **649** |
| R41.0 | Disorientation, unspecified | 467 (17.7%) | 285 (26.0%) | 81 (9.1%) | 101 (15.6%) |
| R41.8 | Other symptoms and signs involving cognitive functions and awareness | - | - | - | - |
| R41.80 | Other symptoms and signs involving cognitive functions and awareness | 1,018 (38.6%) | 318 (29.0%) | 526 (59.2%) | 174 (26.8%) |
| R41.88 | Other symptoms and signs involving cognitive functions and awareness | 120 (4.6%) | 62 (5.7%) | 31 (3.5%) | 27 (26.8%) |
| R41.9 | Unspecified symptoms and signs involving cognitive functions and awareness | - | - | - | - |
| F06.7 | Mild cognitive disorder | 427 (16.2%) | 173 (15.8%) | 104 (11.7%) | 150 (23.1%) |
| F00.2 | Dementia in Alzheimer’s disease, atypical or mixed type (coded with G30.8) | 21 (0.8%) | 9 (0.8%) | 5 (0.6%) | 7 (1.1%) |
| F00.9 | Dementia in Alzheimer’s disease, unspecified (coded with G30.8) | 42 (1.6%) | 12 (1.1%) | 19 (2.1%) | 11 (1.7%) |
| F01.5 | Vascular dementia, with and without behavioural disturbance | - | - | - | - |
| F01.9 | Vascular dementia, unspecified | 77 (2.9%) | 28 (2.6%) | 22 (2.5%) | 27 (4.2%) |
| F02.3 | Dementia in Parkinson’s disease | 7 (0.3%) | 2 (0.2%) | 3 (0.3%) | 2 (0.3%) |
| F02.8 | Dementia in other diseases classified elsewhere, with and without behavioural disturbance | 14 (0.5%) | 2 (0.2%) | 7 (0.8%) | 5 (0.8%) |
| F03 | Unspecified dementia | 352 (13.4%) | 145 (13.2%) | 89 (10.0%) | 118 (18.2%) |
| G20 | Parkinson’s disease | 186 (7.1%) | 91 (8.3%) | 45 (5.1%) | 50 (7.7%) |
| G30.8 | Other Alzheimer’s disease | 2 (0.1%) | 2 (0.2%) | - | - |
| G30.9 | Alzheimer’s disease, unspecified | 58 (2.2%) | 18 (1.6%) | 21 (2.4%) | 19 (2.9%) |
| G31.0 | Circumscribed brain atrophy | 2 (0.1%) | 2 (0.2%) | - | - |
| G31.02 | Frontal lobe dementia | 12 (0.5%) | 2 (0.2%) | 6 (0.7%) | 4 (0.6%) |
| G31.8 | Other specified degenerative diseases of nervous system (Lewy Body Disease) | 14 (0.5%) | 7 (0.6%) | 5 (0.6%) | 2 (0.3%) |
| G319 | Possible major vascular neurocognitive disorder | 37 (1.4%) | 26 (2.4%) | 4 (0.5%) | 7 (1.1%) |
| ^a^ Includes diagnosis and pre-existing diagnosis categories. | | | | | |

| **NATIONAL AMBULATORY CARE REPORTING SYSTEM** | | | | | |
| --- | --- | --- | --- | --- | --- |
| **ICD-10** | **Definition** | **Total**  **No. (%)** | **Before ICU**  **No. (%)** | **During ICU**  **No. (%)** | **After ICU**  **No. (%)** |
| **Depressive** | | **15,216** | **10,567** | **74** | **4,575** |
| F31.3 | Bipolar affective disorder, current episode mild or moderate depression | 82 (0.5%) | 78 (0.7%) | - | 4 (0.1%) |
| F31.4 | Bipolar affective disorder, current episode severe depression without psychotic symptoms | 15 (0.1%) | 6 (0.1%) | - | 9 (0.2%) |
| F31.5 | Bipolar affective disorder, current episode severe depression with psychotic symptoms | 2 (0.0%) | 2 (0.0%) | - | - |
| F31.6 | Bipolar affective disorder, current episode mixed | 6 (0.0%) | 5 (0.1%) | 1 (1.4%) | - |
| F31.9 | Bipolar affective disorder, current episode unspecified | 881 (5.8%) | 588 (5.6%) | 17 (23.0%) | 276 (6.0%) |
| F32.0 | Mild depressive episode | 89 (0.6%) | 48 (0.5%) | 3 (4.1%) | 38 (0.8%) |
| F32.1 | Moderate depressive episode | 48 (0.3%) | 25 (0.2%) | - | 23 (0.5%) |
| F32.2 | Severe depressive episode without psychotic symptoms | 921 (6.1%) | 565 (5.4%) | 4 (5.4%) | 352 (7.7%) |
| F32.3 | Severe depressive episode with psychotic symptoms | 55 (0.4%) | 53 (0.5%) | 4 (5.4%) | 2 (0.0%) |
| F32.4 | Depressive disorder, single episode, in partial remission | - | - | - | - |
| F32.5 | Depressive disorder, single episode, in the full remission | - | - | - | - |
| F32.8 | Other depressive episodes | 72 (0.5%) | 25 (0.2%) | 2 (2.7%) | 45 (1.0%) |
| F32.9 | Depressive episode unspecified | 4,313 (28.4%) | 3,048 (28.8%) | 45 (60.8%) | 1,220 (26.7%) |
| F33.0 | Recurrent depressive disorder, current episode mild | 1 (0.0%) | - | - | 1 (0.0%) |
| F33.1 | Recurrent depressive disorder, current episode moderate | 394 (2.6%) | 184 (1.7%) | - | 210 (4.6%) |
| F33.2 | Recurrent depressive disorder, current episode severe without psychotic symptoms | 329 (2.2%) | 201 (1.9%) | - | 128 (2.8%) |
| F33.3 | Recurrent depressive disorder, current episode severe with psychotic symptoms | 27 (0.2%) | 8 (0.1%) | - | 19 (0.4%) |
| F33.4 | Recurrent depressive disorder, currently in remission | 10 (0.1%) | 4 (0.0%) | - | 6 (0.1%) |
| F33.8 | Recurrent depressive disorder, other | 110 (0.7%) | 107 (1.0%) | - | 2 (0.1%) |
| F33.9 | Recurrent depressive disorder, unspecified | 981 (6.5%) | 845 (8.0%) | - | 136 (3.0%) |
| F34.1 | Dysthymia | 480 (3.2%) | 273 (2.6%) | - | 207 (4.5%) |
| F34.8 | Other persistent mood disorder | - | - | - | - |
| F34.9 | Persistent mood disorders, unspecified | 2 (0.0%) | 2 (0.0%) | - | - |
| F38.0 | Other single mood disorders | - | - | - | - |
| F38.1 | Other recurrent mood disorders | 2 (0.0%) | 2 (0.0%) | - | - |
| F38.8 | Other specified mood disorders | 1 (0.0%) | 1 (0.0%) | - | - |
| F39 | Unspecified mood disorder | 320 (2.1%) | 239 (2.3%) | - | 81 (1.8%) |
| F41.2 | Mixed anxiety and depressive disorder | 147 (1.0%) | 109 (1.0%) | 2 (2.7%) | 36 (0.8%) |
| F99 | Mental disorder, not elsewhere specified | 6,182 (40.63%) | 4,329 (41.0%) | 5 (6.8%) | 1,848 (40.4%) |
| **Anxiety** | | **4,397** | **3,395** | **16** | **986** |
| F40.0 | Agoraphobia | 100 (2.3%) | 93 (2.7%) | - | 7 (0.7%) |
| F40.1 | Social Phobias | 156 (3.6%) | 111 (3.3%) | - | 45 (4.6%) |
| F40.2 | Specific (isolated) Phobia | 2 (0.1%) | 2 (0.1%) | - | - |
| F40.8 | Other Phobic Anxiety Disorder | 1 (0.0%) | 1 (0.0%) | - | - |
| F40.9 | Phobic Anxiety Disorder, Unspecified | 1 (0.0%) | - | - | 1 (0.1%) |
| F41.0 | Panic Disorder | 174 (4.0%) | 137 (4.0%) | 2 (12.5%) | 35 (3.6%) |
| F41.1 | Generalized Anxiety Disorder | 1,314 (30.0%) | 981 (28.9%) | 1 (6.3%) | 332 (33.7%) |
| F41.8 | Other Specific Anxiety Disorder | 29 (0.7%) | 20 (0.6%) | - | 9 (0.9%) |
| F41.9 | Unspecified Anxiety Disorder | 93 (2.7%) | 2,153 (63.4%) | 13 (81.3%) | 604 (61.3%) |
| **Trauma-and-Stressor Related** | | **2,860** | **2,060** | **19** | **781** |
| F43.0 | Acute stress disorder | 464 (16.2%) | 361 (17.5%) | - | 103 (13.2%) |
| F43.1 | Post-traumatic stress disorder | 635 (22.2%) | 480 (23.3%) | 3 (15.8%) | 152 (19.5%) |
| F43.2 | Adjustment disorders | 1,607 (56.2%) | 1,104 (53.6%) | 16 (84.2%) | 487 (62.4%) |
| F43.8 | Other specified trauma-and stressor related disorder | 11 (0.4%) | 10 (0.5%) | - | 1 (0.1%) |
| F43.9 | Unspecified trauma-and stressor related disorder | 205 (7.2%) | 160 (7.8%) | - | 45 (5.8%) |
| **Neurocognitive** | | **3,283** | **2,324** | **268** | **691** |
| R41.0 | Disorientation, unspecified | 586 (17.9%) | 412 (17.7%) | 23 (8.6%) | 151 (21.9%) |
| R41.8 | Other symptoms and signs involving cognitive functions and awareness | 1,055 (32.1%) | 581 (25.0%) | 237 (88.4%) | 237 (34.3%) |
| R41.80 | Other symptoms and signs involving cognitive functions and awareness | 901 (27.4%) | 499 (21.5%) | 220 (82.1%) | 182 (26.3%) |
| R41.88 | Other symptoms and signs involving cognitive functions and awareness | 155 (4.7%) | 82 (3.5%) | 18 (6.7%) | 55 (8.0%) |
| R41.9 | Unspecified symptoms and signs involving cognitive functions and awareness | - | - | - | - |
| F06.7 | Mild cognitive disorder | 198 (6.0%) | 116 (5.0%) | - | 82 (11.9%) |
| F00.2 | Dementia in Alzheimer’s disease, atypical or mixed type (coded with G30.8) | 1 (0.0%) | 1 (0.0%) | - | - |
| F00.9 | Dementia in Alzheimer’s disease, unspecified (coded with G30.8) | 8 (0.2%) | 3 (0.1%) | - | 5 (0.7%) |
| F01.5 | Vascular dementia, with and without behavioural disturbance | - | - | - | - |
| F01.9 | Vascular dementia, unspecified | 177 (5.4%) | 174 (7.5%) | 1 (0.4%) | 2 (0.3%) |
| F02.3 | Dementia in Parkinson’s disease | 5 (0.2%) | 4 (0.2%) | - | 1 (0.1%) |
| F02.8 | Dementia in other diseases classified elsewhere, with and without behavioural disturbance | 5 (0.2%) | 3 (0.1%) | - | 2 (0.3%) |
| F03 | Unspecified dementia | 276 (8.4%) | 197 (8.5%) | 5 (1.9%) | 74 (10.7%) |
| G20 | Parkinson’s disease | 910 (27.7%) | 774 (33.3%) | 7 (2.6%) | 129 (18.7%) |
| G30.8 | Other Alzheimer’s disease | - | - | - | - |
| G30.9 | Alzheimer’s disease, unspecified | 43 (1.3%) | 30 (1.3%) | - | 13 (1.9%) |
| G31.0 | Circumscribed brain atrophy | 15 (0.5%) | 10 (0.4%) | - | 5 (0.7%) |
| G31.02 | Frontal lobe dementia | 13 (0.4%) | 8 (0.3%) | - | 5 (0.7%) |
| G31.8 | Other specified degenerative diseases of nervous system (Lewy Body Disease) | 25 (0.8%) | 20 (0.9%) | 1 (0.4%) | 4 (0.6%) |
| G319 | Possible major vascular neurocognitive disorder | 45 (1.4%) | 44 (1.9%) | - | 1 (0.1%) |

| **PHYSICIAN CLAIMS** | | | | | |
| --- | --- | --- | --- | --- | --- |
| **ICD-9** | **Definition** | **Total**  **No. (%)** | **Before ICU**  **No. (%)** | **During ICU**  **No. (%)** | **After ICU**  **No. (%)** |
| **Depressive** | | **99,829** | **68,482** | **2,543** | **28,804** |
| 296.2 | Major depressive disorder, single episode  *Mild, moderate, severe without mention of psychotic behaviour, severe specified as with psychotic behaviour, in partial or unspecified remission, in full remission, unspecified* | 965 (1.0%) | 617 (0.9%) | 6 (0.2%) | 342 (1.2%) |
| 296.3 | Major depressive disorder, recurrent episode  *Mild, moderate, severe without mention of psychotic behaviour, severe specified as with psychotic behaviour, in partial or unspecified remission, in full remission, unspecified* | 1,808 (1.8%) | 1,326 (1.9%) | 7 (0.3%) | 475 (1.7%) |
| 296.5 | Bipolar I disorder, most recent episode (or current)  *Mild depression, moderate depression, severe without mention of psychotic behaviour, severe specified as with psychotic behaviour* | 138 (0.1%) | 106 (0.2%) | 1 (0.0%) | 31 (0.1%) |
| 296.6 | Bipolar I disorder, most recent episode (or current) mixed | 207 (0.2%) | 171 (0.3%) | - | 36 (0.1%) |
| 296.8 | Atypical depressive disorder | 1,012 (1.0%) | 791 (1.2%) | 23 (0.9%) | 198 (0.7%) |
| 296.9 | Other specified episodic mood disorder  Unspecified episodic mood disorder | 2,525 (2.5%) | 1,737 (2.5%) | 26 (1.0%) | 762 (2.7%) |
| 298 | Other non-organic depressive psychoses | 13,876 (13.9%) | 9,454 (13.8%) | 556 (21.9%) | 3,866 (13.4%) |
| 300.4 | Dysthymic disorder | 4,678 (4.7%) | 2,693 (3.9%) | 186 (7.3%) | 1,799 (6.3%) |
| 309.0 | Adjustment disorder with depressed mood | 12,629 (12.7%) | 7,541 (11.0%) | 541 (21.3%) | 4,547 (15.8%) |
| 309.0 | Adjustment disorder with depressed mood | 602 (0.6%) | 459 (0.7%) | 8 (0.3%) | 135 (0.5%) |
| 309.1 | Prolonged depressive reaction | 488 (0.5%) | 348 (0.5%) | 1 (0.0%) | 139 (0.5%) |
| 311 | Depressive disorder, not elsewhere classified | 62,331 (62.4%) | 44,285 (64.7%) | 1,199 (47.2%) | 16,847 (58.5%) |
| **Anxiety** | | **50,841** | **35,533** | **756** | **14,552** |
| 300 | Anxiety, dissociative and somatoform disorders | 27,676 (54.4%) | 19,894 (56.0%) | 299 (39.6%) | 7,483 (51.4%) |
| 300.0 | Anxiety states  *Anxiety state unspecified, panic disorders without agoraphobia, generalized anxiety disorder, other anxiety states* | 16,703 (32.9%) | 11,903 (33.5%) | 184 (24.3%) | 4,616 (31.7%) |
| 300.2 | Phobic disorders  *Phobia unspecified, agoraphobic with panic disorder, agoraphobia without mention of panic attacks, social phobia, other isolated or specific phobias* | 98 (0.2%) | 79 (0.2%) | - | 19 (0.1%) |
| **Trauma-and-Stressor Related** | | **5,727** | **3,723** | **155** | **1,849** |
| 308 | Acute reaction to stress | 5,074 (88.6%) | 3,323 (89.3%) | 147 (94.8%) | 1,604 (86.8%) |
| 308.0 | Predominant disturbance of emotions | 508 (9.9%) | 257 (6.9%) | 25 (16.1%) | 226 (12.2%) |
| 308.1 | Predominant disturbance of consciousness | 449 (7.8%) | 80 (2.2%) | 82 (52.9%) | 287 (15.5%) |
| 308.2 | Predominant psychomotor disturbance | 35 (0.6%) | 15 (0.4%) | - | 20 (1.1%) |
| 308.3 | Other acute reactions to stress | 219 (3.8%) | 175 (4.7%) | - | 44 (2.4%) |
| 308.4 | Mixed disorders as reaction to stress | 27 (0.5%) | 17 (0.5%) | - | 10 (0.5%) |
| 308.9 | Unspecified acute reaction to stress | 256 (4.5%) | 179 (4.8%) | 28 (18.1%) | 49 (2.7%) |
| 309.2 | Adjustment disorder with mixed anxiety and depressed mood | 78 (1.4%) | 43 (1.2%) | - | 35 (1.9%) |
| 309.3 | Adjustment disorder with disturbance of conduct | 19 (0.3%) | 5 (0.1%) | 1 (0.7%) | 13 (0.7%) |
| 309.4 | Adjustment disorder with mixed disturbance of emotions and conduct | 26 (0.5%) | 19 (0.5%) | 1 (0.7%) | 6 (0.3%) |
| 309.8 | Other specified adjustment reactions | 240 (4.2%) | 162 (4.4%) | 3 (1.9%) | 75 (4.1%) |
| 309.9 | Unspecified adjustment reaction | 293 (5.1%) | 171 (4.6%) | 3 (1.9%) | 119 ( |
| **Neurocognitive** | | **6,720** | **3,237** | **222** | **3,261** |
| 290.4 | Vascular dementia | 522 (7.8%) | 250 (7.7%) | 3 (1.4%) | 269 (8.3%) |
| 294.1 | Dementia in conditions classified elsewhere | 2,096 (31.2%) | 920 (28.4%) | 9 (4.1%) | 1,167 (35.8%) |
| 331 | Other cerebral degenerations | 3,760 (56.0%) | 1,853 (57.2%) | 202 (91.0%) | 1,705 (52.3%) |
| 331.0 | Alzheimer’s disease | 412 (6.1%) | 230 (7.1%) | 4 (1.8%) | 178 (5.5%) |
| 331.1 | Frontotemporal dementia | 43 (0.6%) | 19 (0.6%) | 2 (0.9%) | 22 (0.7%) |
| 331.8 | Other cerebral degeneration | 277 (4.1%) | 81 (2.5%) | 7 (3.2%) | 189 (5.8%) |
| 331.9 | Cerebral degeneration, unspecified | 57 (0.9%) | 54 (1.7%) | - | 3 (0.1%) |
| 332.0 | Parkinson’s disease | 346 (5.2%) | 215 (6.6%) | 8 (3.6%) | 123 (3.8%) |
| 780.97 | Altered mental status | - | - | - | - |
