# Supplementary material for: Association between delirium in the intensive care unit and subsequent neuropsychiatric disorders
Source: Crit Care. 2020 Jul 31;24:476. doi: 10.1186/s13054-020-03193-x (PMC7393876; doi:10.1186/s13054-020-03193-x)
Supplement: Supplementary file 2 — Additional file 2. Characteristics of Those With and Without Pre-Existing Neuropsychiatric Disorders. The data presented in additional file 2 compare characteristics of patients with and without pre-existing neuropsychiatric disorders from five years prior to ICU admission. [file 13054_2020_3193_MOESM2_ESM.docx]

Additional File 2. Characteristics of Those With and Without Pre-Existing Neuropsychiatric Disorders

| **Characteristic** | **With Pre-Existing Disorders**  **(n=6,442)** | **Without Pre-Existing Disorders**  **(n=4,033)** | **p-Value** |
| --- | --- | --- | --- |
| **Patient Characteristics on ICU Admission** | | | |
| Age, median (IQR) years | 57 (44-68) | 60 (46-71) | <0.001 |
| Male sex, No. (%) | 3,293 (51.1) | 2,744 (68.0) | <0.001 |
| Reason for ICU Admission, No. (%)^a^ |  |  |  |
| Medical | 4,284 (67.3) | 2,172 (54.9) | <0.001 |
| Surgical | 1,322 (20.8) | 1,221 (30.9) |  |
| Neurological | 368 (5.8) | 205 (5.2) |  |
| Trauma | 389 (6.1) | 356 (9.0) |  |
| Comorbidities, No. (%)^b^ |  |  |  |
| AIDS | 28 (0.4) | 6 (0.2) | 0.012 |
| Acute Myocardial Infarction | 350 (5.4) | 270 (6.7) | 0.001 |
| Cancer | 603 (9.4) | 587 (14.6) | <0.001 |
| Cerebrovascular Disease | 298 (4.6) | 201 (5.0) | 0.40 |
| Congestive Heart Failure | 656 (10.2) | 435 (10.8) | 0.33 |
| Chronic Obstructive Pulmonary  Disease | 1,232 (19.1) | 699 (17.3) | 0.023 |
| Dementia | 149 (2.3) | 4 (0.1) | <0.001 |
| Diabetes | 890 (13.8) | 518 (12.8) | 0.15 |
| Diabetes + Complications | 1,055 (16.4) | 694 (17.2) | 0.24 |
| Hemiplegia or Paraplegia | 165 (2.6) | 90 (2.2) | 0.29 |
| Metastatic Cancer | 603 (9.4) | 167 (4.1) | 0.002 |
| Mild Liver Disease | 473 (7.3) | 196 (4.9) | <0.001 |
| Moderate/Severe Liver Disease | 244 (3.8) | 104 (2.6) | 0.001 |
| Peptic Ulcer Disease | 203 (3.2) | 153 (3.8) | 0.077 |
| Peripheral Vascular Disease | 243 (3.8) | 228 (5.7) | <0.001 |
| Renal Disease | 350 (5.4) | 207 (5.1) | 0.50 |
| Rheumatoid Disease | 92 (1.4) | 61 (1.5) | 0.73 |
| Charlson Comorbidity Score, median (IQR) | 1 (0-3) | 1 (0-3) | 0.004 |
| SOFA Score on ICU admission, median (IQR) | 6 (4-9) | 6 (3-9) | 0.006 |
| APACHE II Score on ICU admission, median (IQR) | 19 (14-24) | 18 (13-23) | <0.001 |
| Glasgow Coma Scale score on ICU admission, median (IQR) | 14 (11-15) | 15 (13-15) | <0.001 |
| **Interventions Received in ICU** | | | |
| Invasive Mechanical Ventilation | 4,287 (66.5) | 2,535 (62.9) | <0.001 |
| Noninvasive Mechanical Ventilation | 865 (13.4) | 569 (14.1) | 0.32 |
| Continuous Renal Replacement Therapy | 292 (4.5) | 181 (4.5) | 0.91 |
| Vasoactive medications | 2,677 (41.6) | 1,770 (43.9) | 0.019 |
| **Hospital Characteristics** | | | |
| ≥ 20 ICU Beds, No. (%) | 3,580 (55.6) | 2,161 (53.6) | 0.046 |
| ≥ 600 Hospital Beds, No. (%) | 4,016 (62.3) | 2,404 (59.6) | 0.005 |
| Teaching hospital, No. (%) | 5,317 (82.5) | 3,304 (81.9) | 0.42 |
| ≥ 24 hours in ICU after ready for ICU discharge | 2,500 (38.8) | 1,505 (37.3) | 0.13 |
| **Patient Characteristics on ICU Discharge** | | | |
| Length of ICU Stay, median (IQR) days | 4.0 (2.3-7.7) | 4.1 (2.3-7.6) | 0.76 |
| Delirium, No. (%) | 3,813 (59.2) | 1,799 (44.6) | <0.001 |
| ^a^ Mutually exclusive categories; 156 patients missing data  ^b^ Non-mutually exclusive categories  Abbreviations: AIDS=Auto-Immune Deficiency Syndrome, APACHE II Score=Acute Physiology and Chronic Health Evaluation II Score, ICU=Intensive Care Unit, SOFA=Sequential Organ Failure Assessment. | | | |
