# Supplementary material for: Association between delirium in the intensive care unit and subsequent neuropsychiatric disorders
Source: Crit Care. 2020 Jul 31;24:476. doi: 10.1186/s13054-020-03193-x (PMC7393876; doi:10.1186/s13054-020-03193-x)
Supplement: Supplementary file 3 — Additional file 3. Unadjusted Risk Ratios for Delirium, Patient Characteristics and Neuropsychiatric Disorders. The data presented in additional file 3 present detailed unadjusted risk ratios for delirium, patient characteristics and each neuropsychiatric disorder. [file 13054_2020_3193_MOESM3_ESM.docx]

Additional File 3. Unadjusted Risk Ratios for Delirium, Patient Characteristics and Neuropsychiatric Disorders

| **Variables** | **Risk Ratios (95% CI)** | | | | | |
| --- | --- | --- | --- | --- | --- | --- |
|  | **Any Neuropsychiatric Disorder** | **Depressive Disorders** | **Anxiety Disorder** | **Trauma-and-Stressor Related Disorders** | **Neurocognitive Disorders** | |
| Delirium | 1.36 (1.20-1.54) | 1.45 (1.20-1.74) | 1.31 (1.08-1.60) | 1.14 (0.79-1.63) | 2.19 (1.57-3.09) | |
| Age | 0.99 (0.99-1.00) | 0.99 (0.98-0.99) | 0.99 (0.98-0.99) | 0.98 (0.97-0.99) | 1.03 (1.02-1.04) | |
| Female | 1.23 (1.08-1.40) | 1.14 (0.94-1.38) | 1.15 (0.93-1.41) | 1.47 (1.01-2.11) | 1.21 (0.86-1.68) | |
| ICU Admission Reason |  |  |  |  |  | |
| Medical | 1.00 (reference) | 1.00 (reference) | 1.00 (reference) | 1.00 (reference) | 1.00 (reference) | |
| Surgical | 0.94 (0.80-1.09) | 1.02 (0.81-1.27) | 0.89 (0.69-1.13) | 1.58 (1.04-2.40) | 0.62 (0.40-0.95) | |
| Neurological | 1.85 (1.48-2.26) | 1.86 (1.30-2.55) | 1.59 (1.06-2.27) | 2.53 (1.26-4.63) | 2.34 (1.36-3.77) | |
| Trauma | 1.65 (1.37-1.97) | 1.98 (1.51-2.56) | 1.87 (1.40-2.45) | 2.12 (1.18-3.62) | 1.43 (0.84-2.30) | |
| APACHE II Score | 1.00 (1.00-1.01) | 1.00 (0.99-1.01) | 0.99 (0.98-1.00) | 0.99 (0.96-1.01) | 1.05 (1.03-1.07) | |
| Charlson Comorbidity Index | 0.98 (0.95-1.02) | 0.98 (0.93-1.03) | 0.93 (0.88-0.99) | 0.88 (0.78-0.99) | 1.10 (1.01-1.19) | |
| Glasgow Coma Scale | 0.95 (0.94-0.97) | 0.95 (0.93-0.98) | 0.97 (0.95-1.00) | 0.94 (0.90-1.00) | 0.89 (0.86-0.92) | |
| ICU Length of Stay ≥ 7 days | 1.40 (1.23-1.59) | 1.54 (1.27-1.85) | 1.39 (1.12-1.70) | 1.82 (1.25-2.61) | 1.40 (0.99-1.95) | |
| Last SOFA score | 1.00 (0.97-1.03) | 1.01 (0.96-1.06) | 0.95 (0.90-1.00) | 1.02 (0.93-1.11) | 1.07 (0.99-1.15) | |
| Transfer Delay ≥ 24 hours | 1.13 (0.99-1.28) | 1.09 (0.90-1.31) | 0.92 (0.75-1.13) | 1.12 (0.77-1.61) | 1.52 (1.10-2.10) | |
| Invasive Mechanical Ventilation | 1.18 (1.03-1.35) | 1.31 (1.08-1.61) | 1.22 (0.99-1.52) | 1.53 (1.04-2.33) | 1.35 (0.95-1.94) | |
| Continuous Renal Replacement Therapy | 1.25 (0.94-1.60) | 1.50 (1.02-2.11) | 0.87 (0.50-1.39) | 1.38 (0.59-2.70) | 1.80 (0.93-3.11) | |
| Non-Invasive Mechanical Ventilation | 0.89 (0.73-1.07) | 0.75 (0.55-1.00) | 0.97 (0.72-1.28) | 0.64 (0.33-1.14) | 0.78 (0.45-1.26) | |
| Vasoactive Medications | 1.08 (0.95-1.22) | 1.08 (0.89-1.29) | 1.00 (0.82-1.22) | 0.85 (0.59-1.23) | 1.33 (0.96-1.85) | |
| ≥ 20 ICU Beds | 1.24 (1.09-1.41) | 1.28 (1.06-1.55) | 1.19 (0.97-1.46) | 1.30 (0.90-1.89) | 1.35 (0.97-1.90) | |
| Teaching Hospital | 1.15 (0.97-1.37) | 1.15 (0.90-1.49) | 1.23 (0.94-1.65) | 1.37 (0.84-2.39) | 1.33 (0.86-2.19) | |
|  | | | | | |  |
