# Supplementary material for: Association between delirium in the intensive care unit and subsequent neuropsychiatric disorders
Source: Crit Care. 2020 Jul 31;24:476. doi: 10.1186/s13054-020-03193-x (PMC7393876; doi:10.1186/s13054-020-03193-x)
Supplement: Supplementary file 4 — Additional file 4. Adjusted Models for Delirium and Neuropsychiatric Disorders. The data presented in additional file 4 present detailed models for delirium and each neuropsychiatric disorder. [file 13054_2020_3193_MOESM4_ESM.docx]

Additional File 3. Adjusted Models for Delirium and Neuropsychiatric Disorders

| **Variables** | **Risk Ratios (95% CI)** | | | | | |
| --- | --- | --- | --- | --- | --- | --- |
|  | **Any Neuropsychiatric Disorder** | **Depressive Disorders** | **Anxiety Disorder** | **Trauma-and-Stressor Related Disorders** | **Neurocognitive Disorders** | |
| Delirium | 1.14 (0.98-1.33) | 1.16 (0.92-1.45) | 1.16 (0.92-1.47) | 0.82 (0.53-1.28) | 1.59 (1.08-2.35) | |
| Age | 1.00 (0.99-1.00) | 0.99 (0.98-1.00) | 0.99 (0.99-1.00) | 0.98 (0.97-1.00) | 1.04 (1.02-1.05) | |
| Female | 1.34 (1.18-1.53) | 1.27 (1.04-1.54) | 1.26 (1.02-1.56) | 1.58 (1.08-2.29) | 1.31 (0.93-1.84) | |
| ICU Admission Reason |  |  |  |  |  | |
| Medical | 1.00 (reference) | 1.00 (reference) | 1.00 (reference) | 1.00 (reference) | 1.00 (reference) | |
| Surgical | 0.99 (0.84-1.16) | 1.08 (0.85-1.37) | 0.90 (0.69-1.17) | 1.74 (1.10-2.73) | 0.62 (0.39-0.97) | |
| Neurological | 1.56 (1.22-1.95) | 1.61 (1.09-2.29) | 1.39 (0.90-2.06) | 1.97 (0.92-3.88) | 1.49 (0.83-2.55) | |
| Trauma | 1.55 (1.24-1.92) | 1.68 (1.21-2.30) | 1.53 (1.08-2.15) | 1.41 (0.71-2.71) | 2.01 (1.10-3.53) | |
| APACHE II Score | 1.00 (0.98-1.01) | 1.00 (0.98-1.02) | 0.99 (0.97-1.01) | 0.99 (0.96-1.03) | 1.00 (0.97-1.03) | |
| Charlson Comorbidity Index | 1.02 (0.98-1.06) | 1.04 (0.99-1.10) | 1.00 (0.93-1.07) | 0.96 (0.84-1.08) | 1.06 (0.97-1.16) | |
| Glasgow Coma Scale | 0.97 (0.95-0.99) | 0.98 (0.95-1.01) | 0.99 (0.95-1.03) | 0.96 (0.90-1.03) | 0.90 (0.85-0.95) | |
| ICU Length of Stay ≥ 7 days | 1.25 (1.07-1.45) | 1.36 (1.08-1.72) | 1.29 (1.00-1.66) | 2.20 (1.38-3.48) | 0.91 (0.62-1.34) | |
| Last SOFA score | 1.00 (0.97-1.03) | 1.01 (0.97-1.06) | 0.96 (0.90-1.01) | 1.03 (0.94-1.12) | 1.04 (0.96-1.12) | |
| Transfer Delay ≥ 24 hours | 1.08 (0.95-1.22) | 1.06 (0.87-1.28) | 0.86 (0.69-1.06) | 1.10 (0.75-1.60) | 1.43 (1.02-1.98) | |
| Invasive Mechanical Ventilation | 0.92 (0.78-1.08) | 0.96 (0.76-1.23) | 1.01 (0.78-1.30) | 1.00 (0.63-1.63) | 0.96 (0.63-1.48) | |
| Continuous Renal Replacement Therapy | 1.17 (0.87-1.54) | 1.31 (0.86-1.92) | 0.86 (0.48-1.44) | 1.43 (0.58-3.08) | 1.62 (0.80-2.98) | |
| Non-Invasive Mechanical Ventilation | 0.89 (0.72-1.08) | 0.79 (0.57-1.07) | 1.03 (0.75-1.39) | 0.75 (0.37-1.38) | 0.66 (0.37-1.08) | |
| Vasoactive Medications | 0.98 (0.85-1.14) | 0.95 (0.77-1.18) | 0.98 (0.78-1.24) | 0.71 (0.46-1.10) | 0.92 (0.63-1.34) | |
| ≥ 20 ICU Beds | 1.03 (0.88-1.22) | 1.03 (0.81-1.31) | 0.96 (0.74-1.23) | 1.01 (0.64-1.61) | 1.10 (0.73-1.67) | |
| Teaching Hospital | 1.01 (0.82-1.25) | 0.96 (0.70-1.31) | 1.11 (0.80-1.56) | 1.15 (0.63-2.19) | 1.06 (0.63-1.86) | |
|  | | | | | |  |
