# Supplementary material for: Association between delirium in the intensive care unit and subsequent neuropsychiatric disorders
Source: Crit Care. 2020 Jul 31;24:476. doi: 10.1186/s13054-020-03193-x (PMC7393876; doi:10.1186/s13054-020-03193-x)
Supplement: Supplementary file 5 — Additional file 5. Sensitivity Analyses Between Delirium and No Delirium/one calendar day/two or more calendar days of Delirium in the Intensive Care Unit. The data presented in additional file 5 is the outputs of a sensitivity analysis between delirium and no delirium/one calendar day/two or more calendar days of delirium in the ICU. [file 13054_2020_3193_MOESM5_ESM.docx]

Additional File 5. Sensitivity Analyses Between Delirium and No Delirium/one calendar day/two or more calendar days of Delirium in the Intensive Care Unit

|  | **Adjusted Risk Ratio (95% Confidence Interval)^a^** | | | |
| --- | --- | --- | --- | --- |
| **Outcome** | **Ever Delirium^b^** | **One Calendar Day of Delirium** | **Two or More Calendar Days of Delirium^b^** | |
| Any Neuropsychiatric Disorder | 1.14 (0.98-1.33) | 1.08 (0.89-1.30) | 1.19 (1.00-1.42) | |
| Depressive | 1.16 (0.92-1.45) | 1.15 (0.86-1.52) | 1.16 (0.89-1.50) | |
| Anxiety | 1.16 (0.92-1.47) | 1.04 (0.75-1.41) | 1.26 (0.96-1.65) | |
| Trauma-and-Stressor  Related | 0.82 (0.53-1.28) | 0.65 (0.32-1.20) | 0.93 (0.57-1.54) | |
| Neurocognitive | 1.59 (1.08-2.35) | 0.95 (0.53-1.63) | 2.14 (1.40-3.28) | |
| ^a^ Adjusted for: age, sex, ICU admission reason (medical, surgical, neurological, trauma), APACHE II Score, Charlson Comorbidity Score, Glasgow Coma Scale, transfer delay ≥ 24 hours, invasive mechanical ventilation (yes/no), non-invasive mechanical ventilation (yes/no), continuous renal replacement therapy (yes/no), vasoactive medications (yes/no), ≥ 20 ICU beds, teaching hospital (yes/no), ICU length of stay (categorized as < 7 days vs. ≥ 7 days), and last SOFA score.  ^b^ Reference group: Never had delirium. | | | |  |
